# The association between minor recurrent aphthous stomatitis (RAS), children’s poor oral condition, and underlying negative psychosocial habits and attitudes towards oral hygiene

**DOI:** 10.1186/s12887-018-1094-y

**Published:** 2018-04-13

**Authors:** S. Tecco, S. Sciara, G. Pantaleo, A. Nota, A. Visone, S. Germani, E. Polizzi, E. F. Gherlone

**Affiliations:** 1 Dental School, Vita-Salute San Raffaele University and IRCCS San Raffaele, Via Olgettina 58, I-20132 Milan, Italy; 2 UniSR-Social.Lab [Research Methods], Faculty of Psychology, Vita-Salute San Raffaele University, Milan, Italy; 3 University of Tor Vergata, Rome, Italy; 4 Center for Oral Hygiene and Prevention, Dental School, Vita-Salute San Raffaele University and IRCCS San Raffaele, Milan, Italy

**Keywords:** Stomatitis, Aphthous, Caries, Dental, Psychosocial Attitudes, Habits, and Oral Health, Psychosocial Prevention and Control, Applied Social Psychology

## Abstract

**Background:**

Minor Recurrent Aphthous Stomatitis (RAS) represents a disease which is very difficult to prevent. This case-control study focused on possible associations between minor Recurrent Aphthous Stomatitis in children, their oral health, and underlying behavioral indexes of children’s attitudes and habits pertaining to (home) oral hygiene, with the further goal of enabling the dentist to prevent these specific kind of lesions, both from a clinical and a broader psychosocial perspective.

**Methods:**

Four hundred one school-children (5–10 years old) in Milan (Italy) were submitted to an intra-oral examination, and interviewed with the aid of a brief psychosocial questionnaire.

**Results:**

At the clinical level, statistically significant associations were observed between the presence of decayed teeth and minor Recurrent Aphthous Stomatitis (*Odds Ratio*: 3.15; *95% CI*: lower limit 1.06; upper limit: 9.36; *Z-test*: 2.07, *p* = 0.039; *Chi-square* = 4.71, *p* = 0.030), and between the Decayed Missing or Filled Teeth (DMFT) index and minor aphthous stomatitis (*Odds Ratio*: 3.30; *95% CI*: lower limit 1.13; upper limit: 9.67; *Z-test* = 2.18, *p* = 0.029; *Chi-square* = 5.27; *p* = 0.022), both results pointing to a significant increase—by circa 3 times—in the risk of developing minor Recurrent Aphthous Stomatitis in children exposed to the two above-identified factors (i.e., the presence of decayed teeth and a clearly compromised oral condition, as signaled by the DMFT index), if compared with the risk run by their non-exposed counterparts. At the psychosocial level of analysis, statistically significant associations were observed (1) between children’s practice of spontaneously brushing teeth when not at home and a comparatively lower (i.e. better) Decayed Missing or Filled Teeth index (*Chi-square*: 8.95; *p* = 0.011), and (2) between receiving parental aid (e.g., proper brushing instructions) while practicing home oral hygiene and a significantly reduced presence of decayed teeth (*Chi-square* = 5.40; *p* = .067; *Spearman’s Rho*, *p* = .038). Further, significant associations were also observed between children’s reported severity of dental pain and both (a) the presence of decayed teeth (*Chi-square* = 10.80; *p* = 0.011), and (b) children’s (poor) oral health condition as expressed by the Decayed Missing or Filled Teeth index (*Chi-square* = 6.29; *p* = 0.043). Interestingly, specific lifestyles and social status, showed no systematic association to other clinical or psychological/psychosocial indices.

**Conclusions:**

These systematic relations suggest that, in the presence of Recurrent Aphthous Stomatitis in pediatric patients, the dentist should carefully monitor children for potential carious lesions, implement protocols of prevention to control Recurrent Aphthous Stomatitis disease in children affected by caries, and also be particularly aware of the right or wrong habits children may acquire in the course of continued social exchange with their caregivers and peers.

**Electronic supplementary material:**

The online version of this article (10.1186/s12887-018-1094-y) contains supplementary material, which is available to authorized users.

## Background

Minor Recurrent Aphthous Stomatitis (minor RAS) represents a disease which is very difficult to prevent. It is a painful oral mucosal condition characterized by recurrent, multiple, small, round ulcers, surrounded by erythematous haloes, with a necrotic base [1, 2].

A minor RAS episode is characterized by a group of 2–5 ulcers with a size of less than 1 cm in diameter. In a period of about 4–14 days the lesions disappear, and there aren’t scars [3].

The scientific literature does not clarify the etiology of minor RAS, but it seems to be related to multifactorial causes [3], such as allergy, trauma [4], genetic predisposition, endocrine disorders, stress and anxiety, and microbiota in the mouth [5, 6]. Minor RAS episodes are often treated with drugs, topical and systemic, that are considered palliative. This treatment is mainly aimed to decrease inflammation and pain [3].

With respect to the *psychosocial* side of the issue, there is mounting empirical evidence showing that studies and considerations about oral health status, origins and consequences cannot be limited—any longer—only to clinical issues. Both actual and perceived oral health conditions do in fact heavily affect young patients’ quality of life [7], especially in the form of social acceptance by peers [8]. Outcomes of this kind become especially significant when one considers that research has repeatedly shown that parents tend to underestimate the influence of poor oral conditions on their children’s emotional and social quality of life [9]. This happens while oral health and dento-facial appearance actually play, instead, a pivotal role in contributing to children’s self-esteem and proper social functioning—a finding established long time ago in social psychology and clearly relating to social acceptance, also known as the ‘what is beautiful is good’ stereotype [10, 11]. Children’s self-confidence and smooth social functioning depend heavily on healthy (facial) appearance [8, 9]. Monitoring children’s behavioral attitudes, habits and practices of (home) oral hygiene becomes, thus, of paramount importance, especially because young patients’ attitudes, habits and (good/bad) practices of oral hygiene may all critically affect their actual oral status and, in turn, prompt or hinder the manifestation of precarious oral conditions, such as—for instance—those leading to unwanted episodes of minor RAS.

The importance of scrutinizing the joint influence—and relative outcomes—of both clinical and psychosocial factors in health-related interventions has been recently documented both by research conducted in the area of dentistry and dental hygiene [12, 13] as well as by research run in other apparently more distant, yet crucial clinical and psychosocial research areas. [14].

Aim of the study: By focusing on the role of specific clinical and psychosocial variables, considered as potential risk factors (i.e., children’s right or wrong behavioral attitudes and habits of home oral hygiene, children’s lifestyle, the presence of systemic diseases, and children’s general oral hygiene conditions), this study examined possible associations between the above variables and the insurgence of episodes of minor RAS. The ultimate goal of the study was to gather and analyze systematic information to help dentists, hygienists and clinicians in developing useful strategies for preventing the manifestation of such lesions, both from a clinical and a broader psychosocial perspective.

## Method

This observational case-control study analyzed the associations between oral aphthosis, specific behavioral indexes of psychosocial attitudes, habits, and practices of oral hygiene, lifestyle, systemic diseases, and oral health in a sample of 401 School children, aged mostly 6–10 years, in Milan, Italy. Data were collected during a prevention project of celiac disease, implemented through an agreement between the City Administration of Segrate (Milan, Italy) and the Vita-Salute San Raffaele University (Milan, Italy). The STROBE checklist was used to report the main findings of the study. The protocol was approved by the Ethic Committee of the Vita-Salute San Raffaele University (Milan, Italy). Data were recorded in full accordance with ethical principles, including the World Medical Association Declaration of Helsinki.

Data were collected between 15th November 2014 and 21st January 2015. The team of operators was composed by 4 dentists, 2 dental hygienists and 8 dental students from San Raffaele Hospital, Vita-Salute San Raffaele University, Milan. The team visited all the children of the School, during several classes. In each class, the operators first gave a short talk to the children about the importance of oral health, nutrition and proper oral hygiene. Then, once obtained parental consent, children were visited by our team of dentists. After this careful intra-oral examination, the children underwent a psychosocial interview [15] and asked a few questions, on a questionnaire-basis, about the behavioral component of their personal attitudes and habits towards oral hygiene and related feelings (e.g. *“Do you usually brush your teeth, also when you are not at home?”*, *“When brushing your teeth, does someone help and instruct you?”*, *“Did you ever had teeth ache?”*, etc.), their lifestyles (e.g. the habit of having an usual snack at school, of practicing sports, possessing and using a personal tablet, etc.), and eventually the presence of systemic diseases (food intolerance, celiac disease).

Case/control ascertainment consisted in the detection of minor RAS, based on clinical observation and a careful conversation with the children about the frequency of episodes (see Additional file 1 for details).

The following variables were collected.

*Clinical indices*:

- Enamel hypoplasia;
- Enamel Hypoplasia of incisors;
- Enamel Hypoplasia of molars;
- Decayed teeth;
- Filled teeth;
- Decayed, Missing and Filled teeth, leading to the DMFT index;
- Fissures sealings.

*Psychosocial* and *medical* indices and predictors:

- The behavioral component of attitudes towards home oral hygiene and related feelings, operationalized in form of interview questions with categorical multiple-choice answering format (“Do you usually brush your teeth, also when you are not at home?” [1 = no, never; 2 = sometimes; 3 = always], “When brushing your teeth, does someone help and instruct you?” [1 = no; 2 = sometimes; 3 = yes, regularly], “Did you ever had teeth ache?” [1 = no, never; 2 = yes, just sometimes/a bit; 3 = yes, I did]);
- Possession of a personal tablet, as an indicator of socio-economic status and, presumably, of family wealth;
- Habit of eating snacks at School, and type of snacks more frequently eaten;
- Presence of food intolerance(s);
- Diagnosis of celiac disease;
- Regular practice of sports activities;
- Topical fluoride treatments at the dentist.

All variables were collected and evaluated with the explicit agreement of all operators, both in the case of clinical observations, and with respect to questionnaire answers (see Additional file 1 for details). The sample included all the children in the School.

Data were handled at patient level (see Additional file 1 for details).

Statistical analyses were performed using the SPSS software. The independence vs. association of categorical and ordinal variables was analyzed using the chi-square test and, in some cases, supplemented by the Spearman Rho non-parametric correlation coefficient. Further, odds ratios (*OR*s), 95% confidence intervals, and Z-tests were computed to further illuminate, in terms of possible risk factors, the observed associations. The threshold for statistical significance was set at *p* = 0.05 for all statistical tests.

## Results

Among 425 potentially eligible children, all enrolled in the School, 401 children obtained parent consent to receive dental visit, and were thus considered eligible participants for the study. Among the eligible children, 14 cases with minor RAS and 387 controls were ascertained. A percentage of 3.5% of all eligible children (i.e., 14 children out of 401) represents the prevalence of minor RAS in our sample. Missing data or inconsistent data were not disclosed.

Sample’s main demographics (age and gender) are reported in Table 1.

**Table 1:** Age and gender distribution for the whole sample

	Gender		
Age	Male n (%)	Female n (%)	Total n (%)
5 years old	1 (0.25%)	0	1 (0.25%)
6 years old	45 (11.2%)	33 (8.2%)	78 (19.4%)
7 years old	55 (13.7%)	33 (8.2%)	88 (21.9%)
8 years old	37 (9.2%)	49 (12.2%)	86 (21.4%)
9 years old	37 (9.2%)	41 (10.2%)	78 (19.4%)
10 years old	26 (6.4%)	40 (10%)	66 (16.4%)
11 years old	0	3 (0.7%)	3 (0.7%)
12 years old	1 (0.25%)	0	1 (0.25%)
Total	202 (50.4%)	199 (49.6%)	401 (100%)

Further, with respect to the whole sample and on a purely descriptive level, we observed the following general sample characteristics:

- The majority of children practiced regularly some sports, with 34.2% of the whole sample (137 children out of 401) practicing more than one sports, and only 15.5% of the whole sample (62 children out of 401) practicing no sports at all;
- More than a third of all interviewed children (39.2% of the whole sample, 157 children out of 401) possessed a personal tablet for private use, this indicating a comparatively higher social status in every child out of three;
- Five children out of 401 (1.2% of the whole sample) resulted affected by celiac disease;
- Twenty-five children out of 401 (6.2% of the whole sample) presented food intolerances, mainly due to milk and dairy products (11 children out of 25).

Further, always with respect to the whole sample, and on a purely descriptive level, we observed also the following characteristics pertaining to children’s oral health:

- Prevention appeared to be not very diffused, as 56 children out of 401 (14% of the whole sample) showed sealants on permanent first molars, and only 45 children out of 401 (11.2% of the whole sample) were used to receive professional topical fluoride treatments in the dental office;
- Eighty children out of 401 (20% of the whole sample) had decayed teeth;
- Eighteen children out of 401 (4.5% of the whole sample) had filled teeth;
- Enamel hypoplasia was present in 72 children out of 401 (18% of the whole sample), mostly at the incisive teeth (51 children out of 72 with enamel hypoplasia at the incisive area);
- In our sample of 401 children aged 5–10 years old, prevalence of RAS was 3.5%.

Numbers of participants assigned to each exposure category are reported in Table 2, along with chi-square tests of independence for comparing (sub-)categories of study participants (with Yates’ continuity correction, if intra-cell *n*s < 5).

**Table 2:** Raw numbers of exposed vs. non-exposed children (‘cases’ vs. ‘controls’) observed in each study category with respect to each of the potential risk factors. *P-values* refer to statistical procedures testing for the association between the examined factors and Minor Recurrent Aphthous Stomatitis (RAS). % values are shown in parenthesis

Factors of Exposure	Categories	Number (%) of children among the 387 Controls	Number (%) of children among the 14 Cases	Statistical Significance of Results
Sports Practice	No sports at all	58 (15%)	4 (28.6%)	*p* = 0.32, *n.s.*
	One single sports activity	195 (50.4%)	7 (50%)	
	More than 1 sports activity	134 (34.6%)	3 (21.4%)	
Decayed teeth	No	**311 (80.8%)**	**8 (57.1%)**	***p*** = 0.03***
	Yes	**74 (19.2%)**	**6 (42.9%)**	
Usual snacks at School:				
a) Milky and dairy	No	172 (44.4%)	7 (50%)	*p* = 0.68, *n.s*.
	Yes	215 (55.6%)	7 (50%)	
b) Sugars and carbohydrates	No	84 (21.7%)	1 (7.1%)	*p* = 0.32, *n.s.*
	Yes	300 (77.5%)	13 (92.9%)	
c) Fruits	No	348 (89.9%)	13 (92.9%)	*p* = 0.72, *n.s.*
	Yes	39 (10.1%)	1 (7.1%)	
d) Fruit Juices	No	359 (92.8%)	14 (100%)	*p* = 0.30, *n.s.*
	Yes	28 (7.2%)	0	
e) No snacks	No	382 (98.7%)	14 (100%)	*p* = 0.67, *n.s.*
	Yes	5 (1.3%)	0	
Ownership of a personal Tablet	No	234 (60.6%)	9 (64.3%)	*p* = 0.78, *n.s.*
	Yes	152 (39.4%)	5 (35.7%)	
Diagnosis of Celiac disease	No	381 (98.7%)	14 (100%)	*p* = 0.67, *n.s*.
	Yes	5 (1.3%)	0	
Food Intolerances	No	363 (93.8%)	13 (92.9%)	*p* = 0.89, *n.s.*
	Yes	24 (6.2%)	1 (7.1%)	
Intolerant to what?	Milk and dairy products	10 (41.7%)	1 (100%)	*p* = 0.93, *n.s*.
	Solanaceae	4 (16.7%)	0	
	Hazelnuts	3 (12.5%)	0	
	Eggs	3 (12.5%)	0	
	Fish	2 (8.3%)	0	
	Gluten	2 (8.3%)	0	
Diagnosis of celiac disease in family	No	375 (96.9%)	12 (85.7%)	*p* = .13, *n.s*.
	Yes	12 (3.1%)	2 (14.3%)	
Enamel hypoplasia	No	316 (82.3%)	10 (71.4%)	*p* = 0.30, *n.s*.
	Yes	68 (17.7%)	4 (28.6%)	
Enamel hypoplasia of incisors	No	336 (87.3%)	12 (85.7%)	*p* = 0.86, *n.s*.
	Yes	49 (12.7%)	2 (14.3%)	
Enamel hypoplasia of molars	No	364 (94.5%)	13 (92.9%)	*p* = 0.79, *n.s*.
	Yes	21 (5.5%)	1 (7.1%)	
Filled teeth	No	368 (95.8%)	11 (84.6%)	*p* = 0.22, *n.s*.
	Yes	16 (4.2%)	2 (15.4%)	
DMFT index	DMFT = 0	**294 (76.8%)**	**7 (50%)**	***p*** = 0.02***
	DMFT ≥1	**89 (23.2%)**	**7 (50%)**	
Sealing of teeth	No	327 (84.9%)	12 (85.7%)	*p* = 0.93, *n.s*.
	Yes	54 (14.0%)	2 (14.3%)	
	Sealing of deciduous teeth	4 (1.0%)	0	
Topical fluoride treatments in the dental office	No	342 (88.4%)	12 (85.7%)	*p* = 0.90, *n.s*.
	Yes	43 (11.1%)	2 (14.3%)	
	Don’t know	2 (0.5%)	0	

*** significant at least at *p* < .05; *n.s.*: not significant. Statistically significant patterns of results are displayed in bold

A significant association was observed between the presence of decayed teeth and minor RAS (*Odds Ratio* = 3.15, *95% CI* = lower limit 1.06, upper limit 9.36; *Z-test* = 2.07, *p* = 0.039. A chi-square analysis confirmed the observed result, *Chi-square* = 4.71, *p* = 0.030). In sum, the risk of presenting minor RAS resulted 3.15 times higher for children with decayed teeth than for their non-exposed counterparts.

A significant association was observed also between the DMFT index > 0 and minor RAS (*Odds Ratio* = 3.30, *95% CI* = lower limit 1.13, upper limit 9.67; *Z-test* = 2.18, *p* = 0.029. The observed result was independently confirmed by a chi-square test, *Chi-square* = 5.27, *p* = 0.022). In sum, the risk of presenting minor RAS resulted 3.30 times higher for children with DMFT index > 0 than for their non-exposed counterparts.

At the *psychosocial* level of analysis—i.e. at the level of analysis explicitly relating psychological and clinical outcomes—statistically significant associations were observed between (a) the habit of brushing teeth when not at home and a comparatively lower (i.e. better) DMFT index (Chi-square = 8.95, *p* = 0.011), and between (b) receiving parental aid (e.g., proper instructions) while practicing home oral hygiene and a comparatively reduced presence of decayed teeth (*Chi-square* = 5.40, *p* = .067; *Spearman’s Rho*, *p* = .038).

Further, significant associations were also observed between children’s reporting of severity of sensed dental pain and (a) the presence of decayed teeth (*Chi-square* = 10.80, *p* = 0.011), and (b) children’s oral health condition as expressed by the DMFT index (*Chi-square* = 6.29, *p* = 0.043), respectively.

Lifestyles, social status, and systemic diseases showed no association with clinical or psychological indices (all *p*_*s*_ > .05).

Given the straightforward and plain nature of our research design, and given our declared interest only into primary clinical and psychosocial outcomes, no further subgroup or interaction analyses were performed.

## Discussion

This study aimed to investigate associations between distinct behavioral indexes of psychosocial attitudes, habits, and children’s common practices of (home) oral hygiene, general oral health conditions, and the concomitant presence of minor RAS, with the general aim of better understanding, and possibly also preventing, this disorder both from a clinical and a broader psychosocial vantage point.

First, data showed a statistically significant association between decayed teeth and minor RAS.

Only eight children (2.5%) out of 319 children without decayed teeth were found to have ulcers, while 6 (7.5%) out of 80 children with one or more decayed teeth were found to have ulcers (*p* = .039). The presence of decayed teeth was more frequent in patients with recurrent aphthous compared to healthy subjects. For this reason, the presence of caries seems to predispose more to the development of recurrent aphthous. To be sure, in exposed children the risk of presenting minor RAS resulted to be about 3 times higher for children with decayed teeth than for their non-exposed counterparts (*OR* = 3.15, *p* = 0.039).

Similarly, data showed also the presence of a statistically significant association between DMFT and minor RAS. Only seven (2.3%) out of 301 children with DMFT = 0 were found to have ulcers, while 7 (7.3%) out of 96 of children with DMFT > 0 were found to have ulcers (*p* = .029). Ulcers were significantly more frequent in patients with DMFT > 0 rather than in healthy participants. Again, in exposed children the risk of presenting minor RAS resulted about 3 times higher for children with DMFT > 0 than for their non-exposed counterparts (*OR* = 3.30, *p* = 0.029).

We also predicted and found, at the *psychosocial* level of analysis, two further statistically significant associations between (a) the habit of brushing teeth when not at home and a better DMFT index, and between (b) receiving specific parental aid while practicing home oral hygiene and a minor presence of decayed teeth. As negative behavioral attitudes may easily compromise proper practices of oral hygiene and, in turn, lead to decayed teeth and compromised oral status (e.g., minor RAS), the above findings also point to the need of promoting the acquisition of positive attitudes towards oral hygiene by children, through appropriate strategies based on ad-hoc intervention policies and psychosocial monitoring resources and tools.

By the same token, the significant direct associations between children’s reported severity of dental pain with (a) the actual presence of decayed teeth, and with (b) children’s general deteriorated oral health condition—as testified to by a comparatively lower DMFT index—, both suggest to pay close attention also to the nuances of children’s (un)expressed feelings and sensations— such as, in our case, pain and teeth ache.

No statistical association between ulcers and filled teeth was detected. This outcome gave us a further confirmation that caries in the active phase (teeth decayed, not the filled teeth), rather than the presence of filled teeth in itself, seem to predispose to the appearance of recurrent aphthous.

Data from this sample did not detect significant associations between RAS and celiac disease, food intolerances, and further indicators of lifestyle such as the customary consumption of snacks at School, the more or less frequent practice of sports, or general social-status as inferred by observing whether children possessed a tablet or not.

In the presence of a direct association between RAS and decayed teeth in pediatric patients, the concomitant presence of recurrent aphthae must suggest particular care in evaluating the occurrence of carious lesions, and also the necessity of implementing protocols of prevention to control RAS disease in children affected by caries.

Given the documented association between the psychosocial behavioral indices of a positive general attitude towards oral hygiene and oral health, and given also the documented association between proper oral-health maintenance instructions received by parents and compromised dental status (DFMT), special attention should be paid also to those *psychological* indices, aspects, and predictors that are able to strengthen children’s *commitment* towards proper hygiene and oral status, on the one hand, and oral health, on the other.

In comparing the results of the present study with those of similar studies and other relevant evidence, we may note that the etiology of RAS ulcers, in the research literature, is still not entirely clear and known. For instance, Sunday et al. [16] conducted research on possible causes of RAS and concluded that the etiology of RAS lesions is still unknown as several local, systemic, immunologic, genetic, allergic, nutritional, and microbial factors have been proposed as causative agents. Also, some medications including immunosuppressive drugs such as calcineurin and mTOR inhibitors have been associated with severe aphthous-like stomatitis. They do not make a direct reference to the presence of cariogenic bacteria, but only to the variation of the saliva composition, of saliva, such as PH, which is certainly a factor that correlates with the presence of caries lesions in the active phase in the oral cavity [17–20].

With respect to nutrition and lifestyles, data analysis did not detect significant associations. In the literature, a study by Tarakji et al. [21] assessed the correlation between eating habits and the occurrence of aphthous stomatitis. Their analysis too suggested that, ‘Dietary habits have no important role in development of RAS but can be playing a minor role in the pathogenesis of RAS either by causing hypersensitivity or by deficiency of some vitamins and minerals’. As a case in point, their study showed that RAS patients eat acidic PH-containing foods, like oranges and lemons, more frequently than controls, and this habit might have initiated RAS lesions as irritation factors—while other patients might have had some form of hypersensitivity to specific food such as yoghurt and tomato, and spicy food. Also, as research has shown how the choice of different toothpastes critically affects oral health [22], even such a simple habit could possibly concur to the development of RAS and, as such, would be worth of further empirical investigation.

A clear limitation of this study concerns the dental visit that, even if done with all of the necessary attention by our operators, was nevertheless performed in a School, i.e. without the dental chair equipment. In addition, some inaccurate data may have been resulted because of the choice to use a questionnaire that, even if administered with all of the necessary care and attention by our operators, may have rendered it difficult to obtain always sincere and precise answers by children. However, despite this manifest difficulty, the sistematicity of our findings suggests that—at least in the case of children’s attitudes and habits—data collection must have had some high degree of reliability.

In this study, we chose to focus on *distal* behavioral indicators of children’s psychosocial habits and attitudes towards oral hygiene, such as their tendency to spontaneously engage in brushing teeth when not at home (i.e., a tendency reflecting children’s general positive attitude towards oral care), or their turning to parents in order to receive concrete advice (i.e., again a tendency revealing children’s behaviorally-anchored positive attitudes, and signaling their confident and purposive social functioning with respect to proper oral hygiene in a supportive social-relational context). However, from a methodological point of view [23], future research will certainly benefit from extending the scope of empirical studies based almost exclusively on the analysis of *behavioral* indices such as those spelled out above. Future research should for instance take into account also specific, more *proximal,* *emotional* and *cognitive* facets of young patient’s attitudes, prejudices, and habits towards oral hygiene, even implicit [24], because patients’ physical and psychological status have clear and direct consequences for perceived quality of life (*QoL*) and well-being, as consistently shown by recent converging research outcomes stemming both from close [12, 13, 25] and distant [26, 27] clinical and psychological research domains. Physical and psychological health are pivotal preconditions for well-balanced and smooth social functioning and social exchange, both at the interpersonal and at the broader societal level [28]. Difficulties children encounter in their everyday oral hygiene should not be considered solely as undesirable obstacles to accurate oral care because, quite on the contrary, moderate difficulties—under proper and specified conditions—can even motivate behavior and related affective responses very strongly, both at the individual and group level [29–33].

Indicators of lifestyle and eating habits can be many, and in this study we have considered only a few. Thus, we cannot exclude that minor RAS might be associated with further lifestyle and/or eating indicators that we did not explicitly consider in this report.

Further, additional clinical and psychosocial studies elucidating the link between RAS/oral hygiene and health-related psychological factors would be extremely relevant and useful in this field. Reserachers could systematically test the idea, for instance, that scanty parental involvement with dental care might be probably *causally* linked to childrens’ poor dental hygiene, and that poor dental hygiene, in turn, could be conducive to both dental decay *and* RAS, in a two-steps causal chain. If such a causative model were true, then it would easily explain why dental decay and (minor) RAS tend, typically, to be correlated with one another in research. Such a correlation would be explained by the fact that these two variables (i.e., dental decay and RAS) would simply represent clinical manifestations of a common causative factor, within the causal chain hypothesized here. Evidently, however, more research is needed to illuminate the issue.

Finally, our results appear to have a generalizability to the school children population.

## Conclusions

The observed association between minor RAS and teeth decay suggests that, in the presence of recurrent aphthous in pediatric patients, closer attention should be paid to the evaluation of potential carious lesions, by implementing protocols of prevention to control RAS disease in children affected by caries, while considering—at the same time—the manifold attitude-related psychosocial indices, markers, and predictors of children’s oral care and, ultimately, oral status and health.

## Additional file

Additional file 1: Case/control ascertainment, collection of variables, handling of data. Additional information about the methods of the study and the variables considered are reported. (DOCX 15 kb)

## Abbreviations

DMFT index: Decayed missing or filled teeth index
RAS: Recurrent aphthous stomatitis
