# Supplementary material for: The association between minor recurrent aphthous stomatitis (RAS), children’s poor oral condition, and underlying negative psychosocial habits and attitudes towards oral hygiene
Source: BMC Pediatr. 2018 Apr 13;18:136. doi: 10.1186/s12887-018-1094-y (PMC5897994; doi:10.1186/s12887-018-1094-y)
Supplement: Supplementary file 1 — Case/control ascertainment, collection of variables, handling of data. Additional information about the methods of the study and the variables considered are reported. (DOCX 15 kb) [file 12887_2018_1094_MOESM1_ESM.docx]

Additional file 1

**Case/control ascertainment**

Clinical observation consisted in the observation of lesions manifested as small rounded or oval ulcers of oral mucosa, covered by a grayish-white pseudomembrane and surrounded by an erythematous halo. The brief conversation was targeted at ascertaining the RAS episodes, i.e. self-limiting and auto-resolving within 4-14 days, without leaving scars, with the appearance of 1-5 ulcers measuring under 1 cm in diameter. The clinical observation involved more than a single operator, and each case ascertainment was established with the agreement of all the operators. Great attention was also paid by operators to what children reported during the brief conversation about the frequency of episodes, especially in the case of younger children. Subjects were then categorized as having or not minor RAS.

**Collection of variables**

More specifically, the clinical observation involved more than a single operator and the case ascertainment was established with the agreement of all operators. The answers to the questionnaire were recorded on the basis of a brief interview with each of the children. The answers were written down only when all operators agreed on their meaning.

To reduce bias due to differences among operators, the survey-form data and the questionnaire were widely discussed and studied by operators. To reduce bias due to differences among operators, the survey-form data and the questionnaire were widely discussed and studied by operators, who gathered to standardize the assessment procedure with respect to each variable. In addition, data sets were completed with the agreement of all operators, and each case was discussed when unclear. Also, questionnaire-based interviews to children were conducted by multiple operators, simultaneously (2-3 operators). Groups of operators were also interchanged.

**Handling of data**

Enamel hypoplasia, enamel hypoplasia of incisors, enamel hypoplasia of molars, diagnosis of celiac disease, presence of food intolerance, possession of personal tablet were treated as dichotomous variables (absence vs. presence). Data about fissure sealings were grouped in the following categories: present, not present, and present at the deciduous teeth. Data about decayed/filled teeth (and the DMFT index) were handled, always at patient level, by grouping together participants with a number of decayed/filled teeth ≥ 1. Data about practicing sports were handled by grouping participants, regardless of the type of sports they practices, into the following three categories: children who did not practice any sports and, therefore, had a rather sedentary life (score = 0); children who practiced only one sports activity and/or devoted to sports only a little part of their free time (score = 1); children who practiced more than one sports activity and were also very active, spending most of their free time with sports (score = 2).

The answers relative to the consumption of a customary snack at School were coded by grouping data into the following 5 categories: milk and dairy products; sugars and carbohydrates; fruits; fruit juices; nothing. The answers relating to food intolerances were handled by grouping data into the following categories: milk and dairy products; solanaceae, hazelnuts, eggs, fish, and gluten. The behavioral component of children’s attitudes and concrete habits towards home oral hygiene were assessed in form of interview questions, and coded by adopting the following categorical multiple-choice answering format for each of the questions, respectively: “When not at home, do you brush your teeth as well?” [1 = no; 2 = sometimes; 3 = always”], “When brushing your teeth, does someone help and instruct you?” [1 = no; 2 = sometimes; 3 = yes, regularly”], “Did you ever had teeth ache?” [1 = no, never; 2 = yes, just sometimes/a bit; 3 = yes, I did”].
